# A patient with polymerase E1 deficiency (*POLE1*): clinical features and overlap with DNA breakage/instability syndromes

**DOI:** 10.1186/s12881-015-0177-y

**Published:** 2015-05-07

**Authors:** Isabelle Thiffault, Carol Saunders, Janda Jenkins, Nikita Raje, Kristi Canty, Mukta Sharma, Lauren Grote, Holly I Welsh, Emily Farrow, Greyson Twist, Neil Miller, David Zwick, Lee Zellmer, Stephen F Kingsmore, Nicole P Safina

**Affiliations:** Division of Clinical Genetics, Childrens Mercy Hospital, 2420 Pershing Road, Suite 421, Kansas City, MO 64108 USA; Center for Pediatric Genomic Medicine, Children’s Mercy Hospital, Kansas City, MO 64108 USA; Department of Pathology and Laboratory Medicine, Childrens Mercy Hospitals, Kansas City, MO 64108 USA; Pediatric Allergy, Asthma and Immunology Clinic, Children’s Mercy Hospitals, Kansas City, MO 64108 USA; Dermatology Clinic, Children’s Mercy Hospitals, Kansas City, MO 64108 USA; Department of Hematology and Oncology, Children’s Mercy Hospitals, Kansas City, MO 64108 USA; Department of Pediatrics, Children’s Mercy Hospitals, Kansas City, MO 64108 USA; University of Missouri, Kansas City School of Medicine, Kansas City, MO USA

**Keywords:** POLE1, FILS syndrome, Immunodeficiency, Dysmorphism, Primordial dwarfism

## Abstract

**Background:**

Chromosome instability syndromes are a group of inherited conditions associated with chromosomal instability and breakage, often leading to immunodeficiency, growth retardation and increased risk of malignancy.

**Case presentation:**

We performed exome sequencing on a girl with a suspected chromosome instability syndrome that manifested as growth retardation, microcephaly, developmental delay, dysmorphic features, poikiloderma, immune deficiency with pancytopenia, and myelodysplasia. She was homozygous for a previously reported splice variant, c.4444 + 3A > G in the *POLE1* gene, which encodes the catalytic subunit of DNA polymerase E.

**Conclusion:**

This is the second family with *POLE1*-deficency, with the affected individual demonstrating a more severe phenotype than previously described.

**Electronic supplementary material:**

The online version of this article (doi:10.1186/s12881-015-0177-y) contains supplementary material, which is available to authorized users.

## Background

Chromosome instability syndromes are a group of inherited conditions associated with chromosomal instability and breakage which includes LIG4 [1,2], Seckel type 1 [3,4], Bloom syndrome [5], Nijmegen breakage syndrome [6-8], and Fanconi anemia. These genetic conditions are characterized by pre and postnatal growth retardation, microcephaly, dysmorphic features and bone marrow failure [9-11]. There are other conditions with overlapping phenotypes including microcephaly, such as Rad50 deficiency [12,13], Cernunnos-XLF syndrome [14] and Warsaw breakage syndrome [15-18] which can complicate molecular diagnosis. FILS syndrome (facial dysmorphism, immunodeficiency, livedo, and short stature) is a recently described condition caused by variants in *POLE1*, encoding the catalytic subunit of polymerase E. We describe the second family with a homozygous variant in *POLE1,* and a more severely affected individual, suggesting a broader phenotypic spectrum for this condition.

## Case presentation

**Figure 1:**
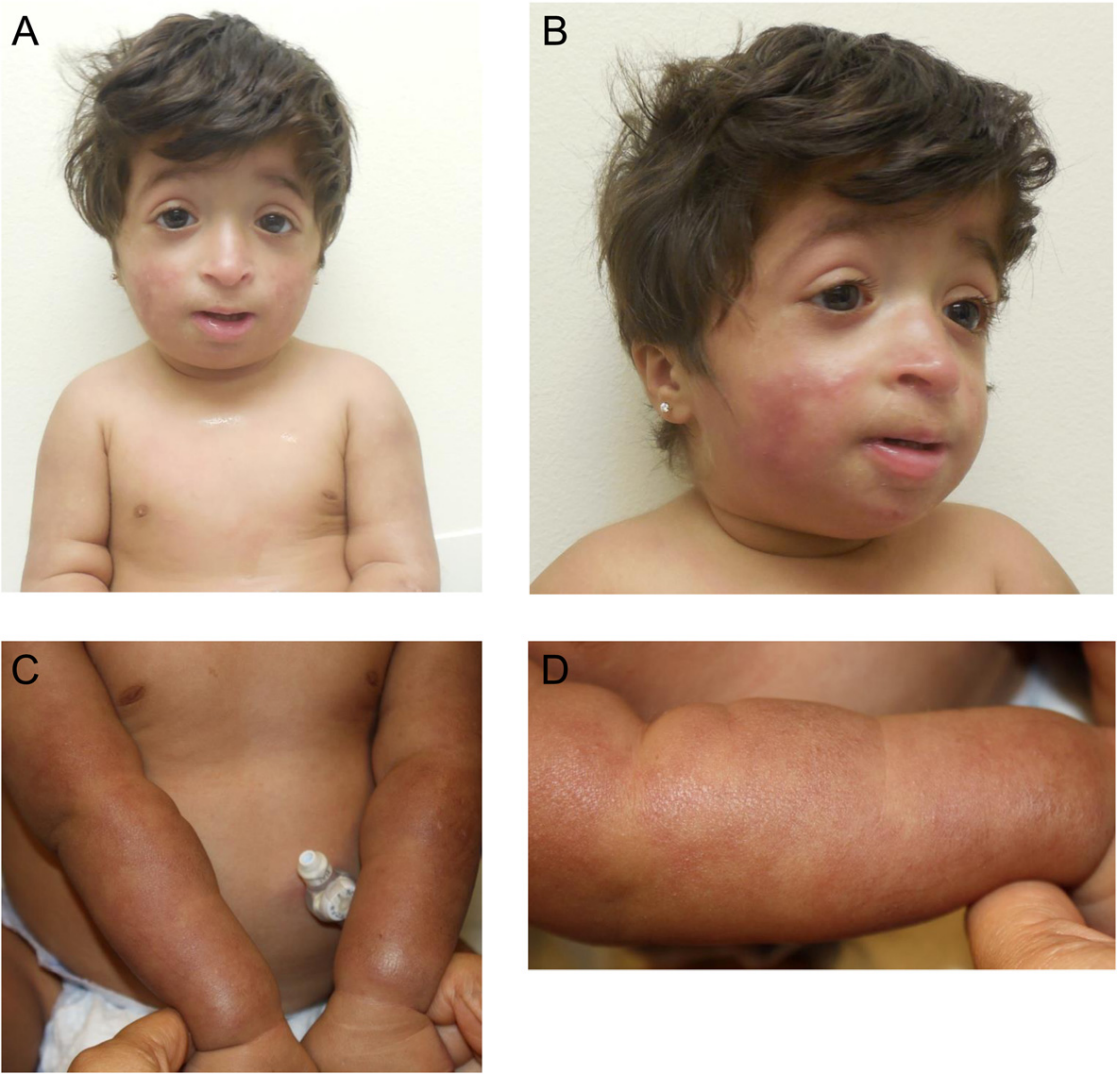
Clinical characteristics of CMH812. Several congenital abnormalities were found, including **(A)** microcephaly, facial dysmorphism (small and bird-like face, malar and mandibular hypoplasia, prominent nasal bridge and columella, downslanting palpebral fissures, small mouth and low set, posteriorly rotated ears) **(B)** short stature with shortened long bones but no evidence of dysplasia or craniosynostosis. No major anomalies were found on imaging of her abdomen, brain, brain vasculature or heart. Genitalia and pubertal development were normal. No malabsorption or pituitary or thyroid insufficiency was found. **(C-D)** Skin findings in CMH812, showing one hypo- and three hyperpigmented patches on the skin. Biopsy of the skin was performed but not diagnostic. Microscopic examination displayed focal parakeratosis and mild spongiosis.

**Figure 2:**
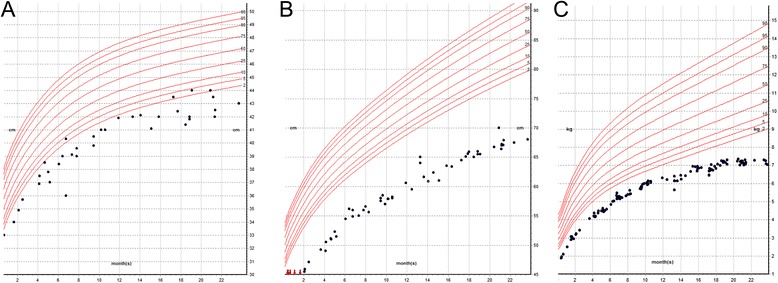
Growth charts of CMH812. The patient is a female with severe intrauterine and postnatal growth retardation head circumference **(A)** [<3rd centile]; weight **(B)** [<2nd centile], length **(C)** [<2nd centile], based on CDC growth chart for girls, age 0–20 months. Growth remained poor postnatally and she was briefly started on growth hormone, which was discontinued due to suspicion for a disorder of DNA repair/instability. At age 15 months she was ~5 SD below the mean for weight and length and ~3-4 SD below the mean for head circumference.

Patient CMH812 is a female infant born to healthy non consanguineous Palestinian parents, weighing 1745 g and measuring 38.1 cm at birth. The pregnancy was complicated by subchorionic bleeding in the first trimester, fetal abnormalities on ultrasound including intrauterine growth restriction, short long bones, suspected skull abnormalities and oligohydramnios. TORCH titers were negative. Amniocentesis revealed normal 46,XX karyotype. She was delivered at 37 weeks gestation by elective C-section secondary to breech presentation. Dysmorphic features noted included malar and mandibular hypoplasia (Figure 1A, B). Initial clinical suspicion was for primordial dwarfism such as Seckel type 1 syndrome, however her microcephaly was not as severe. Over several months, lacy reticular pigmentation was noted of the face and extremities. She had recurrent pruritic papular eruptions and skin findings progressed to include appearance of poikiloderma (Figure 1C, D). Erupted teeth were found to be small and dysplastic. She developed a feeding aversion necessitating a gastrostomy tube. Growth remained poor postnatally (Figure 2). Her motor milestones were delayed but social development was normal.

She suffered chronic rhinosinusitis and pulmonary infections with purulent otitis media. At age 20 months she was admitted to the hospital with pancytopenia, splenomegaly, hepatitis and acute CMV infection. Laboratory data showed mild bone marrow myelodysplasia, normal total B, T, and NK cells, low class switched and non switched memory B cells, and high memory T cells. She had high IgA, normal total IgG and low IgM, IgG2 and IgG4. There was no serologic response to pneumococcal vaccine (Table 1). Lymphocyte response to mitogens was normal but absent to pertussis and candida antigens [11]. Hepatitis and pancytopenia resolved following the CMV infection. Extensive molecular and cytogenetic testing was negative, including array-CGH, telomere length studies, chromosome breakage, and gene sequencing with deletion/duplication analysis for the following genes: *ATM, ATR, BLM, CENPJ, CEP152, CEP63, ERCC6, ERCC8, LIG4, LMNA, MRE11A, NBN, PCNT, RBBP8, RECQL4, SHOX, WRN,* and *ZMPSTE24.*

**Table 1:** **Comparison of clinical features of** ***POLE1*** **patients and other chromosome instability syndromes**

**Clinical features**	**TAR syndrome**	**Ataxia telangiectasia**	**RAD50 deficiency**	**Fanconi anemia**	**Seckel syndrome**	**NBS**	**RS/SC syndrome**	**Warsaw syndrome**	**Cernnos-XLF syndrome**	**Bloom syndrome**	**LIG4 syndrome2**	FILS syndrome*	**CMH812**
MIM **#**	274000	208900	613078	227650	210600	251260	268300 /269000	613398	611291	210900	606593	615139	615139
Microcephaly	−	ν	ν	ν	ν	ν	ν	ν	ν	ν	ν	ν	ν
Malar hypoplasia	−	n.a	ν	−	n.a	n.a	ν	ν	−	ν	n.a	ν	ν
Sloping head	ν	−	n.a	−	ν	ν	ν	ν	ν	−	ν	ν	ν
Palpebral fissures, upslanting	−	−	n.a	−	−	ν	−	ν	−	−	ν	ν	−
Palpebral fissures, down-slanting	−	−	ν	−	ν	−	ν	ν	ν	−	−	ν	ν
Epicanthic folds	−	−	ν	−	−	ν	ν	ν	ν	−	ν	ν	ν
Micrognathia	ν	−	n.a	−	ν	ν	ν	ν	ν	−	−	ν	−
External ear abnormalities	−	−	n.a	ν	ν	ν	ν	ν	ν	ν	−	ν	ν
Long/Large nose	ν	−	ν	−	−	ν	ν	ν	ν	ν	ν	ν	−
Long philtrum	−	−	n.a	−	−	ν	ν	ν	ν	−	−	ν	ν
Cleft palate/high arched palate	−	−	n.a	−	ν	ν	ν	ν	−	−	−	−	−
Polydactily	−	−	n.a	ν	−	ν	−	−	−	ν	−	−	−
Clinodactily	−	−	n.a	−	ν	ν	ν	ν	−	ν	ν	−	ν
Syndactily													
Growth retardation	−	ν	ν	ν	ν	ν	ν	ν	ν	ν	ν	ν	ν
Short stature	ν	ν	n.a	ν	ν	ν	ν	ν	ν	ν	ν	ν	ν
Bone disease or anomalies	ν	−	n.a	ν	ν	−	ν	n.a	ν	−	−	ν	ν
Thumb apasia/radial ray anormalities	ν	−	n.a	ν	ν	−	−/ν	n.a	−	−	ν		−
Skin abnormalities	ν	ν	ν	ν	ν	ν	ν	ν	ν	ν	ν	ν	ν
Mental retardation	ν	ν	ν	ν	ν	ν	ν	ν	ν	−	ν	−	−
Developemental delay	ν	ν	ν	ν	ν	ν	ν	ν	ν	ν	ν	−	ν
Malignancy	ν	ν	−	ν	ν	ν	ν	n.a	ν	ν	ν	−	−
Recurrent infections	−	ν	−	ν	ν	ν	ν	ν	ν	ν	ν	ν	ν
Genital abnormalities	−	−	−	ν	ν	ν	ν	n.a	**−**	−	ν	−	−
Ataxia	−	ν	ν	−	ν	n.a	**−**	n.a	**−**	n.a	n.a	−	−
Brain anomalie/degeneration	CA, CH, aCC, S	CA, CH,T, S	ND	ν	CA, CH, PG, S	ND	S	n.a	PG	n.a	n.a	n.a	−
Endocrine	−	AZ, D, DP, HH	−	HH	D, DP	POF	n.a	n.a	−	AZ, D RF	−	−	−
Immunologic Features													
Pancytopenia	−	n.a	n.a	ν	ν	−	n.a	n.a	ν	−	ν	−	ν
Thrombocytopenia	ν	n.a	−	ν	−	−	n.a	n.a	−	−	−	−	ν
CID	−	ν	−	−	n.a	−	n.a	n.a	ν	n.a	ν	2/14	ν
SCID	−	ν	−	−	ν	−	n.a	n.a	ν	n.a	ν	n.a	-
Neutropenia	−	n.a	n.a	ν	n.a	ν	n.a	n.a	ν	N	ν	n.a	-*
B cell lymphocytopenia	−	ν	N	ν	−	−	n.a	n.a	ν	−	2/17	ν	ν
T cell lymphocytopenia	−	ν	N	ν	ν	ν	n.a	n.a	ν	ν	N	ν	ν
IgA	n.a	↓	N	N	↓	n.a	n.a	n.a	↓	↓	↓	N	↑
IgE	n.a	↓	N	n.a	↓	n.a	n.a	n.a	n.a	N	n.a	N	N
IgG	n.a	↓	N	↓	↓	n.a	n.a	n.a	↓	↓	n.a	↓	↓**
IgM	n.a	↓	N	↓	↓	n.a	n.a	n.a	↓	↓	↓	↓	↓
Anti-pseudomonae	n.a	n.a	N	n.a	↓	n.a	n.a	n.a	ν	n.a	n.a	ν	ν
polysaccaride IgG													
Auto-immunity	n.a	ν	−	n.a	↓	ν	n.a	n.a	1/17	n.a	0/17	−	-
Sister chromatide	n.a	−	n.a	n.a	↑ or N	ν	ν	ν	n.a	ν	n.a	N	N
DNA breakage studies	n.a	ν	ν	ν	ν	ν	ν	ν	ν	ν	n.a	−	N
Radiosensitivity	n.a	ν	ν	ν	ν	ν	ν	ν	ν	ν	ν	−	n.a.
Gene	RBM8A	ATM	RAD50	FANC	ATR	NBS1	ESCO2	DDX11	NHEJ1	BLM	LIG4	POLE1	POLE1
Mode of Inheritance	AR	AR	AR	AR	AR	AR	AR	AR	AR	AR	AR	AR	AR

Legend

n.a; not reported/applicable.

−; negative.

ν; positive.

N; normal range.

↓; decreased.

↑; increased.

NBS: Nijmegen breakage syndrome.

TAR: Thrombocytopenia-absent radius syndrome.

RS: Roberts syndrome.

SC: SC phocomelia syndrome.

aCC; absence of corpus collosum.

AR; autosomal recessive.

AZ; azoospermia.

CA; cerebellar ataxia.

CH; cerebellar hypoplasia.

CID; combined immunodeficiency.

D; diabetes.

DP; delayed puberty.

HH; hypergonadotropic hypogonadism.

NBS; Nijmegen breakage syndrome.

ND; neurodegenerative.

OA; oculomotor apraxia.

PG; polygyria.

POF; primary ovarian failure.

RF; reduced fertility.

S; seizures.

SCID; severe combined immunodeficiency.

T; Tremor.

*consanguineous family reported.

£ Ataxia-telangiectasia-like disorder.

- * Transient pancytopenia associated with CMV infection.

↓** IgG2 and IgG 4 ↓. Total IgG N.

Trio-exome sequencing was performed on CMH812 and her healthy parents (CMH813 & CMH814) following informed consent, and with methods as previously published [19-22]. Variants were filtered to 1% minor allele frequency, then prioritized by the American College of Medical Genetics (ACMG) categorization [23,24], OMIM identity and phenotypic assessment. This individual was homozygous for a splice-site variant, c.4444 + 3A > G, in intron 34 of the *POLE*1 [11]. The parents were both heterozygous carriers (Additional file 1: Table S1). Homozygosity mapper was used to identify intervals of homozygosity and identity by descent segments (Additional file 2: Figure S1) [25].

The c.4444 + 3A > G variant was previously reported in consanguineous French family with three generations of affected members [11]. FILS phenotype was variable but included macrocephaly, recurrent respiratory infections, livedo and telangiectasia, bone dysplasia, short stature, and decreased IgM and IgG. The phenotype was considered similar to that of Bloom syndrome but with normal sister chromatid exchange. Table 1 compares the clinical and cellular features of CMH812 to those of individuals with inherited chromosomal instability and breakage syndromes, as well as the first reported FILS family. Features closely matched those reported in FILS with exceptions of microcephaly and intrauterine growth restriction. Although the *POLE1* variant identified in the present case is the same as previously reported, CMH812 seems to have had more significantly impaired growth and immunity, raising the hypothesis that rare variant(s) in other POLƐ subunits or MMR genes may act as phenotypic modifiers. However, no rare variant were detected in MMR genes, POLE1 interacting proteins or other DNA breakage/instability syndrome genes.

The c.4444 + 3A > G *POLE*1 variant confers abnormal splicing whereby exon 34 is deleted [11] leading to significant decrease in the POLE1 subunit [11]. T- lymphocytes from affected individuals showed a proliferation defect as well as impaired cell cycle progression. The primary function of polymerase Ɛ1 is to synthesize DNA at the leading strand during replication [26,27], however, it is also involved in other cellular processes, including cell cycle progression and DNA repair/recombination [26,27]. Exonucleolytic proofreading and the MMR pathway act to maintain high-fidelity DNA replication and to protect against mutagenesis [28]. Somatic and germline heterozygous missense variants in POLƐ1 have been associated with an increased cancer risk [28-32]. Functional studies in yeast showed that heterozygosity for a pathogenic allele can cause complete MMR deficiency, and that subsequent loss of heterozygosity is not required for the development of POLE-related tumors [28]. Taken together, these findings suggest that *POLE1* carriers are likely to be at increased risk for malignancy due to MMR deficiency.

## Conclusions

In summary, we report a second family with *POLE1*-related disease. The clinical and immunologic features of our patient are reminiscent of LIG4 syndrome, possibly representing the more severe end of an ill-defined clinical spectrum. For this reason, POLE1 deficiency may be a more apt description of this disorder. This report illustrates the cost-effectiveness of trio-exome sequencing as a powerful diagnostic method considering that this family underwent an extensive diagnostic odyssey, with no molecular basis identified prior exome.

### Consent statement

The project was approved by the research ethics committee of the Children’s Mercy Hospitals. Written informed consent was obtained from the patient’s legal guardians for publication of this case report. A copy of the written consent is available for review by the Editor-in-Chief of this journal.

## Additional files

Additonal file 1: Table S1. Trio-Exome sequencing data. Additonal file 2: Figure S1. Homozygosity Mapping Analysis. Homozygosity mapper was used to identify intervals of homozygosity. The homozygosity scores are plotted against the physical position. Red and black bars represent the excess (red) or the shortage (black) of homozygosity **(A)** Eighteen loci of > 1 Mb of homozygosity were identified on seven chromosomes; loci ranged between 1.06 and 6.37 Mb in length. The length of identical by descent segments in genomes of CMH812 parents suggests that they are not closely related. However, they shared several relatively small identical segments of genome which is explained by the shared ancestry broken into pieces by the recombination events and Mendelian laws of inheritance. *POLE1* lies in a 1.1 Mb region of homozygosity on chromosome 12 (chr12:132635257–133702440), which includes 20 genes **(B)**. It is reasonable to hypothesize that the *POLE1* variant, if not the result of a combination of recent origin and chance, owes its origin to a founder who lived several hundred years ago. Consistent with the rarity of the c.4444 + 3A > G variant and the small physical distances between each haplotype marker in relationship to *POLE1* gene (1.1 Mb); the size of the shared haplotype may have been broken into smaller segments due to genetic drift. This assumption could explain that the same variant (c.4444 + 3A > G) has been found in two apparently unrelated FILS families.

## Abbreviations

ACMG: American College of Medical Genetics
aCGH: Array of comparative genomic hybridization
DNA: Deoxyribonucleic acid
FILS: Facial dysmorphism immunodeficiency livedo and short stature
IUGR: Intrauterine growth restriction
LIG4: DNA ligase IV
Mb: Megabase pair
MMR: DNA mismatch repair
NK: Natural Killer
OMIM: Online Mendelian Inheritance in Man
NICU: Neonatal intensive-care unit
PCR: Polymerase chain reaction
POLE1: DNA polymerase E1
TAR: Thrombocytopenia absent radii syndrome
TORCH: Toxoplasmosis, other infections, rubella, cytomegalovirus (CMV), and herpes simplex virus (HSV). The “other infections” usually include syphilis, hepatitis B, coxsackie virus, Epstein-Barr virus, varicella-zoster virus, and human parvovirus
SD: Standard deviation
SNP: Single nucleotide polymorphism
XLF: XRCC4-like factor
