# Supplementary material for: A patient with polymerase E1 deficiency (POLE1): clinical features and overlap with DNA breakage/instability syndromes
Source: BMC Med Genet. 2015 May 7;16:31. doi: 10.1186/s12881-015-0177-y (PMC4630961; doi:10.1186/s12881-015-0177-y)
Supplement: Additonal file 2: Figure S1. — Homozygosity Mapping Analysis. Homozygosity mapper was used to identify intervals of homozygosity. The homozygosity scores are plotted against the physical position. Red and black bars represent the excess (red) or the shortage (black) of homozygosity (A) Eighteen loci of > 1 Mb of homozygosity were identified on seven chromosomes; loci ranged between 1.06 and 6.37 Mb in length. The length of identical by descent segments in genomes of CMH812 parents suggests that they are not closely related. However, they shared several relatively small identical segments of genome which is explained by the shared ancestry broken into pieces by the recombination events and Mendelian laws of inheritance. POLE1 lies in a 1.1 Mb region of homozygosity on chromosome 12 (chr12:132635257–133702440), which includes 20 genes (B). It is reasonable to hypothesize that the POLE1 variant, if not the result of a combination of recent origin and chance, owes its origin to a founder who lived several hundred years ago. Consistent with the rarity of the c.4444 + 3A > G variant and the small physical distances between each haplotype marker in relationship to POLE1 gene (1.1 Mb); the size of the shared haplotype may have been broken into smaller segments due to genetic drift. This assumption could explain that the same variant (c.4444 + 3A > G) has been found in two apparently unrelated FILS families. [file 12881_2015_177_MOESM2_ESM.pptx]

## Slide 1
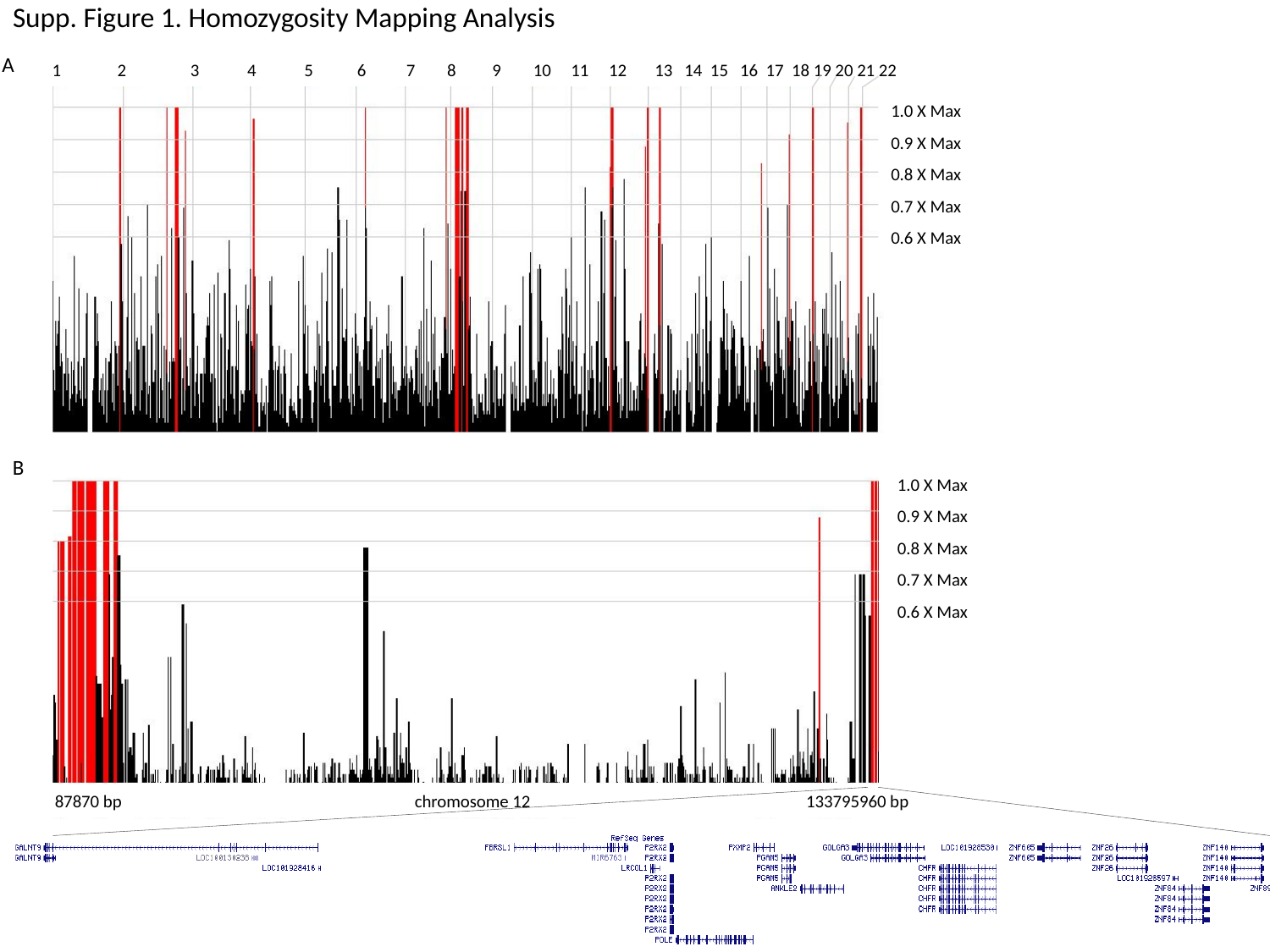

# Supp. Figure 1. Homozygosity Mapping Analysis
A
1 2 3 4 5 6 7 8 9 10 11 12 13 14 15 16 17 18 19 20 21 22
1.0 X Max
0.9 X Max
0.8 X Max
0.7 X Max
0.6 X Max
B
1.0 X Max
0.9 X Max
0.8 X Max
0.7 X Max
0.6 X Max
87870 bp chromosome 12		 133795960 bp
